# Free-Energy Profile of Li-Ion Insertion Reaction in Highly Concentrated Electrolytes: Impact of Destabilized Li-Ion on Electrode Reaction Thermodynamics

**DOI:** 10.1021/acs.jpclett.6c02078

**Published:** 2026-08-09

**Authors:** Saki Sawayama, Misa Yamashita, Kenta Fujii

**Affiliations:** † Graduate School of Sciences and Technology for Innovation, 13150 Yamaguchi University, 1-16-2 Tokiwadai, Ube, Yamaguchi 755-8611, Japan

## Abstract

Understanding the Li-ion insertion reaction at graphite electrodes as a chemical reaction requires a thermodynamic perspective, namely elucidating the underlying free-energy profile. In this study, we quantitatively constructed the free-energy profile of the Li-ion insertion reaction in highly concentrated electrolytes by combining activation thermodynamics derived from electrochemical impedance measurements with experimentally determined solvation free energies of Li^+^. The resulting free-energy profile revealed that the activation free energy (Δ*G* ^⧧^) remains nearly constant across different solvent systems, whereas the solvation free energy of Li^+^ (Δ*G*_Li_) varies significantly depending on solvent properties and dominates the thermodynamics of the electrode reaction. Furthermore, high-energy X-ray total scattering and molecular dynamics simulations showed that Li^+^ forms ion-aggregate structures with anions and solvent molecules, where short-range Li^+^–Li^+^ configurations give rise to electrostatic repulsion, leading to destabilization of Li^+^. These results establish a direct link between solution structure and electrode reaction thermodynamics, providing new insights into the role of Li^+^ solvation in highly concentrated electrolytes.

## Graphical abstract

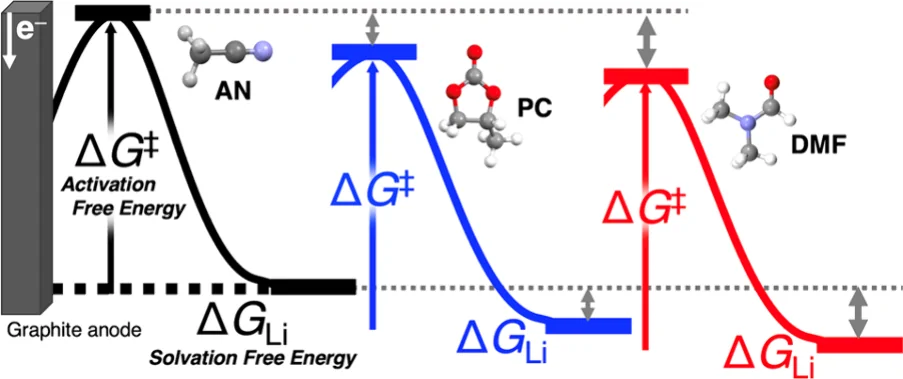

The Li-ion insertion reaction at graphite electrodes is a prototypical electrode process for lithium-ion batteries (LIBs) and should be regarded as a chemical reaction. Understanding a chemical reaction, in general, requires a thermodynamic perspective based on the free energy of the reaction. However, despite its importance, the thermodynamic aspects of this electrode reaction remain insufficiently understood, particularly in complex electrolyte systems such as highly concentrated electrolytes.

Recently, highly concentrated electrolytes, in which large amounts of metal salts are dissolved in a solvent, have attracted considerable attention as a new electrolyte design concept.^1–6^ In such systems, the molar ratio of metal salts to solvent molecules becomes comparable, leading to the near elimination of free solvent in the bulk; consequently, the insufficient number of solvent molecules for solvation results in the formation of ionic aggregates (AGGs), where Li ions continuously associate with counteranions.^7–12^ The formation of such ion-ordered structures characteristic of highly concentrated systems significantly increases the viscosity of the electrolyte compared to dilute systems. However, contrary to the general expectation that high viscosity reduces rate performance, highly concentrated electrolytes often exhibit superior rate capability compared to conventional dilute electrolytes.^2,13,14^ This suggests that the reaction mechanism at the electrode/electrolyte interface may differ from the conventional understanding.

In LIBs, the Li-ion insertion reaction at graphite negative electrodes has been extensively studied, and its reaction kinetics strongly correlates with the desolvation process of Li ions.^15–17^ In particular, for carbonate-based electrolytes widely used as commercial electrolytes, the activation energy for the Li-ion insertion reaction has been reported to be approximately 58 kJ mol^–1^, which is well established to correspond to the desolvation energy of Li ions.^15,16^ This indicates that the reaction is controlled not by the behavior of Li ions within the electrode, but by Li ion–solvent interactions in the electrolyte, namely, solvation. This behavior also applies to highly concentrated systems. Recent systematic studies have demonstrated a positive correlation between Li^+^–solvent interactions and the activation energy for the Li-ion insertion reaction.^18^ This suggests that, even in highly concentrated systems where complex AGGs are formed, the desolvation of solvent molecules coordinated to Li ions within AGGs at the electrode interface kinetically governs the electrode reaction.

Understanding such electrode reaction mechanisms has generally been studied through Arrhenius-type analysis based on the temperature dependence of charge-transfer resistance (*R*_ct_).^15–17,19,20^ The activation energy (*E*_a_) obtained by this method has been widely used as an indicator that strongly correlates with the desolvation process. However, the Arrhenius equation has its origin in molecular collisions in the gas phase, and the activation energy obtained from Arrhenius analysis does not directly provide thermodynamic information on the activation process.^21^ Therefore, a thermodynamic description based on the activation free energy (Δ*G* ^⧧^) is required to achieve a fundamental understanding of chemical reaction processes such as desolvation at the electrode interface, which governs the reaction kinetics.

In this study, we applied transition state theory, which is widely used for ionic reactions in solution (e.g., complexation between metal ions and ligands), to the graphite negative electrode reaction and quantitatively evaluated the activation free energy (Δ*G* ^⧧^) and its enthalpic (Δ*H* ^⧧^) and entropic (*T*Δ*S* ^⧧^) contributions. Furthermore, the solvation free energy of Li ions (Δ*G*_Li_) in the bulk electrolyte was experimentally determined and regarded as the free energy of the initial state, enabling the quantitative construction of the free-energy profile for the Li-ion insertion reaction. By applying this approach to highly concentrated electrolytes, we elucidate the thermodynamic factors governing electrode reactions that are specific to such systems.

We selected acetonitrile (AN), propylene carbonate (PC), and *N*,*N*-dimethylformamide (DMF) as model solvents. Their donor numbers (*D*_N_), a measure of coordination ability toward metal ions, are 14.1 for AN, 15.1 for PC, and 26.6 for DMF.^22^ Highly concentrated electrolytes were prepared by dissolving LiFSA salt in these solvents, with a fixed molar ratio of Li to solvent of 1:2 in all systems [FSA: bis­(fluorosulfonyl)­amide]. The Li salt concentrations (molarity) of these electrolytes correspond to 5.0 M for the AN system, 3.8 M for the PC system, and 4.1 M for the DMF system. Temperature-dependent electrochemical impedance spectroscopy was performed on graphite electrodes in these electrolytes, and the activation thermodynamic parameters (Δ*G* ^⧧^, Δ*H* ^⧧^, and *T*Δ*S* ^⧧^) were determined based on transition state theory, enabling quantitative comparison of solvent-dependent enthalpic and entropic contributions to the reaction barrier. Details of the measurements and analysis are provided in the Supporting Information.

In the Li-ion insertion reaction into graphite electrodes, it is well established that a semicircle is typically observed in the low-frequency region (1–4 Hz) at potentials of 0.4–0.1 V (vs Li/Li^+^), which is attributed to the charge-transfer resistance (*R*_ct_).^15–17,23^ In the present study, all highly concentrated electrolytes exhibited two semicircles in the high- and low-frequency regions, and the diameter of the low-frequency semicircle (approximately 4 Hz, corresponding to *R*_ct_) decreased with increasing temperature (Figure S1). The obtained spectra were well reproduced by fitting with an equivalent circuit model, allowing reliable separation of the interfacial resistance (*R*_int_) and *R*_ct_ at each temperature. The *R*_ct_ is related to the reaction rate constant (*k*) as follows:

1 $$k=\frac{RT}{n^{2}F^{2}CR_{ct}}$$

where *R* is the gas constant, *T* is the absolute temperature, *n* is the number of transferred electrons, *F* is the Faraday constant, and *C* is the concentration of the reacting species. The derivation of this relationship is provided in the Supporting Information. The Eyring equation based on transition state theory is given as follows:^24,25^

$$k=\frac{\kappa k_{B}T}{h}\;\exp\left(-\frac{\Delta G^{⧧}}{RT}\right)=\frac{\kappa k_{B}T}{h}\;\exp\left(-\frac{\Delta H^{⧧}}{RT}\right)\;\exp\left(\frac{\Delta S^{⧧}}{R}\right)$$ 2

where *k*_B_ is the Boltzmann constant, *h* is the Planck constant, and κ is the transmission coefficient. In this study, κ is assumed to be unity (κ = 1). Combining eqs ([Disp-formula eq1]) and ([Disp-formula eq2]), the following expression relating *R*_ct_ to the activation free energy is obtained:

3 $$\ln\left(\frac{k}{T}\right)=-\frac{\Delta H^{⧧}}{R}\frac{1}{T}+\frac{\Delta S^{⧧}}{R}+\ln\left(\frac{k_{B}}{h}\right)$$

Based on this relationship, plotting ln­(*k*/*T*) against 1/*T* (i.e., Eyring plot) yields a straight line, whose slope and intercept correspond to Δ*H* ^⧧^ and Δ*S*, respectively. The resulting Eyring plots are shown in Figure 1a. The plots exhibit good linearity, indicating that the activation process can be consistently described within the transition state framework across different solvents. The Δ*H* ^⧧^, *T*Δ*S* ^⧧^ (at 298 K), and Δ*G* ^⧧^ (=Δ*H* ^⧧^ – *T*Δ*S* ^⧧^) values obtained from the slope and intercept are summarized in Figure 1b. Here, we note that highly concentrated electrolytes based on LiFSA and LiTFSA [TFSA: bis­(trifluoromethanesulfonyl)­amide] are known to form anion-derived solid electrolyte interphases (SEIs) on graphite electrodes;^2,7,11,12^ additional measurements using electrodes preformed with an EC-derived SEI showed essentially identical Eyring behavior (Figure S2), indicating that the activation thermodynamic parameters are insensitive to the nature of the SEI.

**Figure 1:**
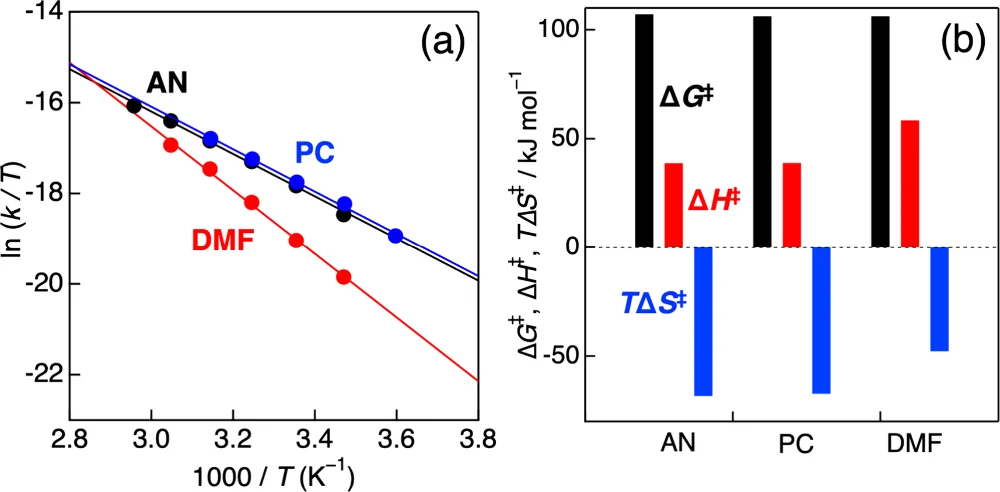
(a) ln­(*k*/*T*) vs 1/*T* plots derived from *R*_ct_ values for the Li-ion insertion reaction at graphite electrodes in highly concentrated LiFSA/AN (black), LiFSA/PC (blue), and LiFSA/DMF (red) electrolytes, measured at a fixed potential of 0.1 V vs Li/Li^+^. (b) The resulting activation thermodynamic parameters, activation free energy (Δ*G* ^⧧^, black), activation enthalpy (Δ*H* ^⧧^, red), and entropy term (*T*Δ*S* ^⧧^, blue).

The activation free energy (Δ*G* ^⧧^) shows similar values across different solvents (107.1 ± 0.4 kJ mol^–1^ for AN, 106.2 ± 0.5 kJ mol^–1^ for PC, and 106.2 ± 0.2 kJ mol^–1^ for DMF), demonstrating that the free energy barrier for the Li-ion insertion reaction in highly concentrated electrolytes is essentially independent of the solvent species. In contrast, the enthalpic and entropic contributions show distinct trends: Δ*H* ^⧧^ is positive and *T*Δ*S* ^⧧^ is negative in all systems, and these values strongly depend on the solvent species.

The Δ*H* ^⧧^ values increase in the order AN (38.8 kJ mol^–1^) ≈ PC (38.9 kJ mol^–1^) < DMF (58.4 kJ mol^–1^), which is consistent with the *D*_N_, a measure of coordination ability toward metal ions, namely AN (14.1) ≈ PC (15.1) < DMF (26.6). The positive Δ*H* ^‡^ reflects the increase in internal energy associated with the desolvation of Li^+^ (i.e., the breaking of Li^+^–solvent interactions) during the charge-transfer process. Accordingly, stronger metal ion–solvent interactions, as indicated by higher *D*_N_ values, result in larger Δ*H* ^⧧^ values. Here, the Δ*H* ^⧧^ values are in good agreement with the activation energies (*E*_a_) obtained from conventional Arrhenius analysis (1/*R*_ct_ ∝ *A* exp­(−*E*_a_/*RT*)), corresponding to the slope of the log­(1/*R*_ct_) versus 1/*T* plots (Figure S3). This result suggests that the *E*_a_ values derived from conventional Arrhenius plots essentially reflect only the internal energy change associated with the charge-transfer process and do not necessarily represent the free energy change, which is the most relevant thermodynamic quantity governing chemical reactions.

In our recent study, we reported that the activation energy of the Li-ion insertion reaction correlates positively with the Li^+^–solvent binding energy (Δ*E*_bind_), based on the temperature dependence of *R*_ct_ (i.e., determination of *E*_a_) for various electrolytes and density functional theory (DFT) calculations.^18^ In the present study, Δ*H* ^⧧^ values were recalculated from previously reported *R*_ct_ data for various electrolyte systems^18^ via transition state theory and plotted against the corresponding Δ*E*_bind_ values, showing a similar linear relationship (Figure S4). These results strongly suggest that the strength of Li^+^–solvent interactions, that is, the difficulty of breaking these interactions, dominantly governs the activation barrier of the electrode reaction in enthalpic terms.

In contrast, the entropic contribution (*T*Δ*S* ^⧧^) takes negative values that compensate for the positive Δ*H* ^⧧^, resulting in similar Δ*G* ^⧧^ values across all systems. In other words, in the rate-determining step of the charge-transfer reaction, solvent effects in highly concentrated electrolytes are not manifested at the level of free energy but are instead reflected in the enthalpic and entropic terms. In general, for ionic reactions in solution, such as complex formation between metal ions and ligands, dissociatively activated pathways involving desolvation often yield positive activation entropies.^25,26^ However, for the Li-ion insertion reaction into graphite electrodes in this study, a negative activation entropy is observed despite desolvation being the rate-determining step. This behavior was also observed in dilute carbonate electrolytes in this study, where Δ*H* ^⧧^ agrees well with the *E*_a_ obtained from conventional Arrhenius analysis, whereas *T*Δ*S* ^⧧^ is negative (Figure S5). These results indicate that the negative entropic contribution is not a feature unique to highly concentrated systems but rather an intrinsic characteristic of electrode reactions.

In electrochemical electrode reactions, the electrode is polarized during charge transfer, and a highly structured and oriented electric double layer is formed at the electrode/electrolyte interface. In contrast, in the bulk electrolyte, that is, in the initial state of the reaction, Li^+^ forms solvated complexes, Li­(solvent)_*n*_^+^ (typically *n* ≈ 4), whose structural organization is lower than that of the electric double layer. As a result, these solvated complexes can diffuse readily in the bulk while undergoing continuous solvent exchange. At the electrode interface, desolvation is expected to increase the degrees of freedom transiently, leading to a positive entropic contribution. However, the high structural ordering of the electric double layer, together with the reorientation of desolvated solvent molecules, significantly restricts molecular freedom. These effects may contribute to the negative *T*Δ*S* ^⧧^ observed in this study. At present, however, it is difficult to quantitatively identify the dominant factor governing the activation entropy term, because multiple interfacial processes may occur simultaneously during Li^+^ insertion. Clarifying the dynamic behavior at the electrode/electrolyte interface will be essential for understanding the microscopic origin of the activation entropy, particularly in highly concentrated electrolytes.

The obtained Δ*G* ^⧧^ values can be combined with the free energy of Li^+^ in the initial state to construct a quantitative free-energy profile for the Li-ion insertion reaction. As a first step toward clarifying the thermodynamic picture of LIB electrode reactions, the initial state is defined here as solvated Li^+^ in the bulk electrolyte, and its free energy (Δ*G*_Li_) is evaluated electrochemically, enabling determination of the free energy profile. The Δ*G*_Li_ in Li-salt-containing electrolytes can be evaluated electrochemically via the redox reaction of ferrocene (Fc), and this approach has been well established.^27,28^ In this study, Δ*G*_Li_ was determined using the same methodology, assuming that solvent effects on the Fc redox reaction are negligible. Figure 2 shows cyclic voltammetry (CV) profiles of the Fc/Fc^+^ redox couple measured in highly concentrated electrolytes (5.0 M LiFSA/AN, 3.8 M LiFSA/PC, and 4.1 M LiFSA/DMF, all with a LiFSA-to-solvent molar ratio of 1:2) containing 1 mM Fc; these data form the basis for evaluating Δ*G*_Li_. The measurements were performed using a three-electrode cell with a Pt working electrode, a Li counter electrode, and a Li reference electrode.

**Figure 2:**
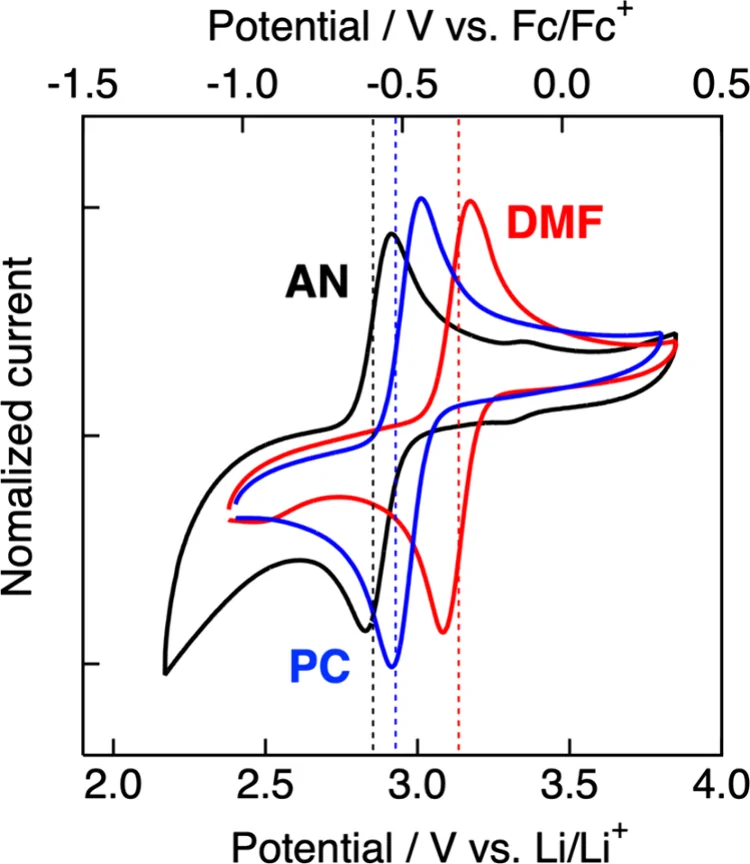
Cyclic voltammograms of the Fc/Fc^+^ redox reaction in highly concentrated LiFSA/AN (black), LiFSA/PC (blue), and LiFSA/DMF (red) electrolytes, showing solvent-dependent shifts in redox potential. Measurements were performed using a three-electrode cell with a Pt working electrode and Li foil as both counter and reference electrodes at a scan rate of 5 mV s^–1^.

For the AN-based electrolyte, the redox potential was determined from the midpoint of the anodic and cathodic peaks in the CV profile, yielding a value of 2.87 V vs Li/Li^+^, corresponding to −0.58 V vs Fc/Fc^+^. In the absence of solvent effects on Fc, the Fc/Fc^+^ redox couple is ideally expected to be located at 3.445 V vs Li/Li^+^ based on the difference in their standard potentials. Therefore, the deviation from this ideal value (3.445 V – 2.87 V = 0.575 V) reflects a shift in the Li/Li^+^ electrode potential arising from the solvation environment. By incorporating this potential shift into the standard Li/Li^+^ potential (−3.045 V vs SHE), the Li/Li^+^ electrode potential in the electrolyte (*E*_Li_) was estimated to be −2.47 V. Furthermore, based on the relationship *E*_Li_ = Δμ_Li_/*nF*, where Δμ_Li_ is the chemical potential of Li ions in solutions, thus corresponding to the solvation free energy of Li^+^, Δ*G*_Li_. The value of Δ*G*_Li_ for the AN system was determined to be −238.3 kJ mol^–1^.

When examining the solvent dependence, the redox potentials shift in the order 2.87 V (AN) < 2.91 V (PC) ≪ 3.11 V (DMF) vs Li/Li^+^, which is consistent with the *D*_N_ values of the solvents: AN (14.1) < PC (15.1) ≪ DMF (26.6). From these values, the Li/Li^+^ electrode potentials (*E*_Li_) were estimated to be −2.47 V (AN), −2.51 V (PC), and −2.71 V (DMF) vs SHE. Using the relationship *E*_Li_ = Δμ_Li_/*nF*, the corresponding Δ*G*_Li_ values were determined to be −238.3 kJ mol^–1^ (AN), −248.0 kJ mol^–1^ (PC), and −261.5 kJ mol^–1^ (DMF). The Δ*G*_Li_ values become increasingly negative from AN to PC and DMF, indicating increased stabilization of Li^+^ with increasing donor number, that is, stronger Li^+^–solvent interactions. Here, it is noteworthy that the Δ*G*_Li_ values obtained for the highly concentrated electrolytes are significantly less negative than those in the corresponding dilute solutions (Figure S6): under the assumption of infinite dilution, Δ*G*_Li_ = −300 kJ mol^–1^ in AN, −365 kJ mol^–1^ in PC, and −336 kJ mol^–1^ in DMF.^29^ This indicates that Li^+^ in highly concentrated electrolytes is substantially destabilized compared to the solvated Li^+^ species formed in dilute solutions, which typically forms as tetra-solvated complexes, Li­(solvent)_4_^+^.

The free-energy profile for the Li-ion insertion reaction at the graphite electrode was constructed by combining the Δ*G*_Li_ and Δ*G* ^⧧^ values obtained for the highly concentrated electrolytes (Figure 3). For the AN system (Figure 3a), Li^+^ in the bulk electrolyte is destabilized by approximately 60 kJ mol^–1^ upon concentration compared to the dilute limit. The destabilized Li^+^ (Δ*G*_Li_ = −238.3 kJ mol^–1^) overcomes a free energy barrier of 107.1 kJ mol^–1^, leading to a transition-state energy of −131.2 kJ mol^–1^ (i.e., Δ*G*_Li_ + Δ*G* ^⧧^), which reflects a state closely associated with the desolvation process. Using the AN system as a reference, the free energy of Li^+^ at the transition state is stabilized by 10.6 kJ mol^–1^ in the PC system and 24.1 kJ mol^–1^ in the DMF system (Figures 3b and 3c). These energy differences closely match those in Δ*G*_Li_ in the bulk electrolyte, which are 9.7 kJ mol^–1^ from AN to PC and 23.2 kJ mol^–1^ from AN to DMF. This correspondence indicates that similar solvent effects operate on Li^+^ both at the transition state and in the initial state in the bulk electrolyte.

**Figure 3:**
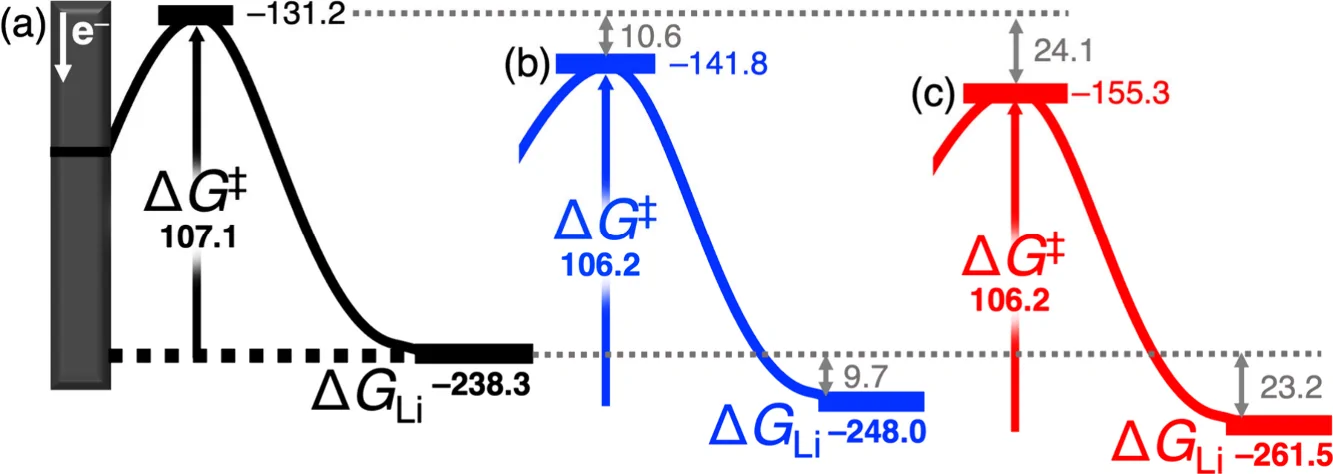
Free-energy profiles for the Li-ion insertion reaction at graphite electrodes in highly concentrated (a) LiFSA/AN, (b) LiFSA/PC, and (c) LiFSA/DMF electrolytes. All energy values are given in kJ mol^–1^.

From these results, it is evident that the activation free energy, Δ*G* ^⧧^ of the Li-ion insertion reaction in highly concentrated electrolytes is largely independent of the solvent species. In contrast, the free energy of Li^+^ in the bulk electrolyte, Δ*G*_Li_ varies significantly and is directly reflected in the free energy at the transition state. These findings indicate that the characteristic solvation free energy of Li^+^ in highly concentrated electrolytes, namely the destabilized Li^+^ state, is a key thermodynamic factor governing the electrode reaction. To elucidate the molecular origin of this destabilization upon increasing concentration, we performed molecular-level structural analysis of the electrolyte solutions using a combination of high-energy X-ray total scattering (HEXTS) and all-atom molecular dynamics (MD) simulations.

Figure 4 shows the X-ray radial distribution functions in differential form, *r* ^2^[*G*(*r*) – 1], obtained from high-energy X-ray total scattering (HEXTS) experiments and all-atom molecular dynamics (MD) simulations. The corresponding X-ray structure factors, *S*(*q*), are shown in Figure S7. Details of the HEXTS measurements and MD simulations are provided in the Supporting Information. For all highly concentrated electrolyte systems, both *G*(*r*) and *S*(*q*) are in good agreement with the experimental data (black circles) and the MD-derived curves. This result indicates that the force field parameters employed in the MD simulations are appropriate for reproducing the actual solution structures. To analyze the local structure around Li^+^, the Li^+^–solvent, Li^+^–FSA^–^, and Li^+^–Li^+^ correlations were extracted from the total radial distribution function *G* ^MD^(*r*), which represents the superposition of all interactions.

**Figure 4:**
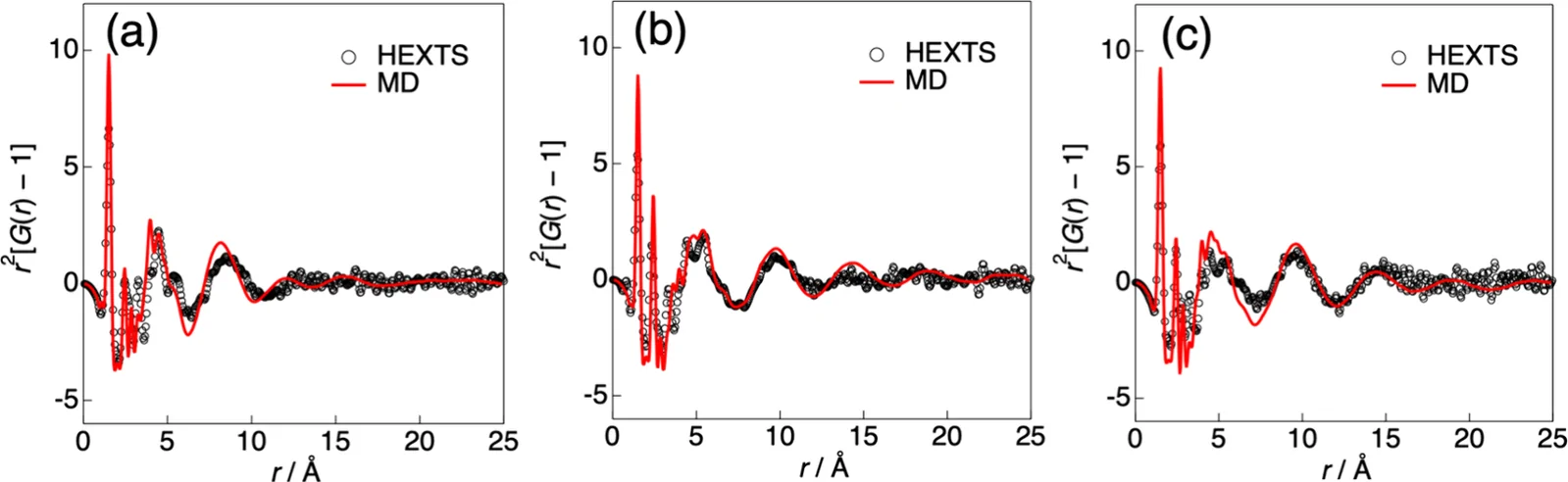
Radial distribution functions in the form of *r* ^2^[*G*(*r*) – 1] obtained from HEXTS experiments (open circles) and MD simulation (solid lines) for highly concentrated (a) LiFSA/AN, (b) LiFSA/PC, and (c) LiFSA/DMF electrolytes.

The pair distribution functions between Li^+^ and the coordinating atoms of each solvent (N for AN, O for PC, and O for DMF), as well as the O atoms of the FSA anion (*g* ^MD^_Li–X_(*r*), where X = N_an_, O_pc_, O_dmf_, and O_fsa_), are shown in Figure S8. In all systems, Li^+^ is coordinated by both solvent molecules and FSA anions. The coordination numbers are approximately 2–2.5 for solvent molecules and 1–2 for FSA anions, giving total coordination numbers of 3.3–3.9. It is well-known that the average coordination number of Li^+^ in dilute solutions, where solvent molecules are present in large excess relative to Li salts, is approximately 4. The reduced coordination numbers observed in the present highly concentrated systems (below 4) are commonly observed under solvent-deficient conditions, where the Li-to-solvent molar ratio is limited (1:2 in this study). In such environments, Li^+^ forms characteristic ionic aggregates (AGGs) based on continuous ion pairing with FSA anions.^7–12^ As a result, the total coordination number becomes smaller than that of the mononuclear solvated complexes, Li­(solvent)_4_^+^, typically observed in dilute solutions.

In the Li^+^–Li^+^ correlation (Figure 5a), a nearest-neighbor peak appears at approximately 3.3 Å, followed by multiple periodic peaks extending to longer distances. The latter long-range periodic features are characteristic of AGG formation, indicating that Li^+^ ions form extended multinuclear complexes linked via anions.^30–33^ Here, we focus on the emergence of the nearest-neighbor peak at ∼3.3 Å. In conventional dilute to moderately concentrated electrolyte systems, Li^+^ typically forms mononuclear solvated complexes or ion pairs through interactions with solvent molecules or counteranions. In such cases, no direct correlation exists between Li^+^ complexes, and therefore no distinct peak appears in the *g* ^MD^_Li–Li_(*r*) function.^30,34^

**Figure 5:**
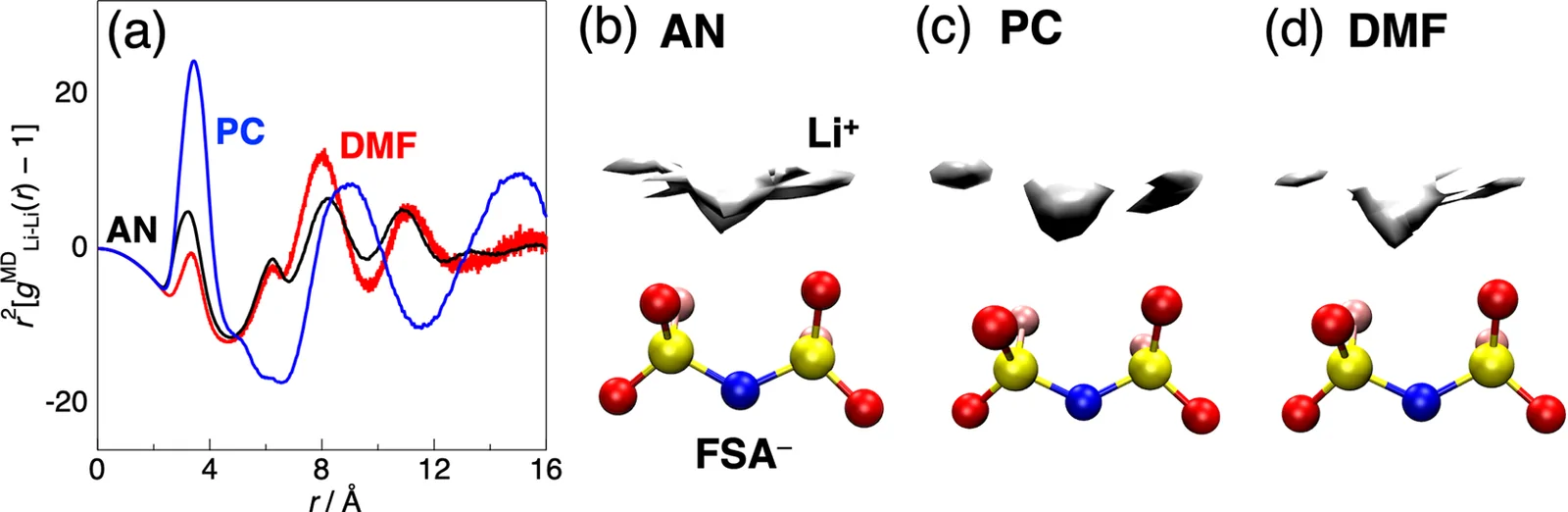
(a) Pair distribution functions for Li^+^–Li^+^ correlations in the form of *r* ^2^[*g* ^MD^_Li–Li_(*r*) – 1] for highly concentrated LiFSA/AN (black), LiFSA/PC (blue), and LiFSA/DMF (red) electrolytes. (b–d) Space distribution functions (SDFs) of Li ions around FSA^–^ in (b) LiFSA/AN, (c) LiFSA/PC, and (d) LiFSA/DMF systems. The clouds indicate isoprobability surfaces of Li ions.

To visualize the origin of the nearest-neighbor peak, space distribution functions (SDFs) of Li^+^ around a central FSA anion were calculated (Figures 5b, 5c, and 5d for the AN, PC, and DMF systems, respectively). The FSA anion possesses high conformational flexibility, allowing the two O atoms of the SO_2_ groups to approach each other.^35–37^ Interaction of Li^+^ with these closely spaced oxygen atoms enables two Li^+^ ions to occupy neighboring positions (gray clouds in the figures), which can be attributed to the Li^+^–Li^+^ correlation peak at ∼3.3 Å. Because Li^+^ ions are positively charged, such short-range Li^+^–Li^+^ configurations are energetically unfavorable due to electrostatic repulsion. Therefore, the short-range Li^+^–Li^+^ configurations within ionic aggregates are suggested to represent a molecular origin of the destabilization of Li^+^ in highly concentrated electrolytes. Within ionic aggregates in highly concentrated electrolytes, electrostatic repulsion between closely spaced Li^+^ ions competes with stabilization arising from interactions between Li^+^ and solvent molecules. As a result of this balance, a destabilized Li^+^ state is formed. This state is also reflected in the stability of the transition state, indicating that the destabilized Li^+^ plays a key thermodynamic role in governing the electrode reaction.

In summary, we constructed a quantitative free-energy profile for the Li-ion insertion reaction at graphite electrodes in highly concentrated electrolytes by combining activation thermodynamics with the solvation free energy of Li^+^. The results revealed that the activation free energy (Δ*G* ^⧧^) is essentially independent of solvent species, while the solvation free energy of Li^+^ (Δ*G*_Li_) varies significantly and governs the thermodynamics of the electrode reaction. Structural analysis further demonstrated that short-range Li^+^–Li^+^ configurations within ionic aggregates induce electrostatic destabilization of Li^+^, providing a molecular origin for the observed thermodynamic behavior. These findings highlight the critical role of Li^+^ solvation thermodynamics in electrode reactions and provide a thermodynamic basis for the rational design of advanced electrolyte systems.

## Supplementary Material



## Notes

Experimental and computational methods, electrolyte compositions for MD simulations, Nyquist plots for highly concentrated electrolytes, ln­(*k*/*T*) vs 1/*T* plots for EC-derived SEI graphite electrode, Arrhenius plots, Δ*H* ^⧧^ vs Δ*E*_bind_ plots, comparison of Δ*H* ^⧧^ and *E*_a_ in dilute carbonate-based electrolytes, Δ*G*_Li_ in dilute and highly concentrated systems, structure factors *S*(*q*), and pair distribution functions *g* ^MD^(*r*) (PDF)
