# Supplementary material for: Free-Energy Profile of Li-Ion Insertion Reaction in Highly Concentrated Electrolytes: Impact of Destabilized Li-Ion on Electrode Reaction Thermodynamics
Source: J Phys Chem Lett. 2026 Aug 9;17(33):9739–45. doi: 10.1021/acs.jpclett.6c02078 (PMC13501948; doi:10.1021/acs.jpclett.6c02078)
Supplement: Supplementary file 1 [file jz6c02078_si_001.pdf]

# Supporting Information

# Free-Energy Profile of Li-ion Insertion Reaction in Highly Concentrated Electrolytes: Impact of Destabilized Li-ion on Electrode Reaction Thermodynamics

*Saki Sawayama,*[a] *Misa Yamashita,* [a] *and Kenta Fujii* [a]*

[a] Graduate School of Sciences and Technology for Innovation, Yamaguchi University, 2-16-1 Tokiwadai, Ube, Yamaguchi 755-8611, Japan

**Corresponding Author**

*E-mail: k-fujii@yamaguchi-u.ac.jp (K.F.)

**Experimental and Computational Methods.**

*Materials.* Lithium bis(fluorosulfonyl)amide (LiFSA) salt (Kanto Chemical, battery grade) was vacuum-dried at 373 K for 24 h prior to use. Acetonitrile (AN, FUJIFILM Wako Pure Chemical, super dehydrated), propylene carbonate (PC, Kishida Chemical), and *N*,*N*-dimethylformamide (DMF, Kishida Chemical) were used as received. Electrolyte solutions were prepared by mixing LiFSA and solvent at a molar ratio of Li salt to solvent = 1:2 in an Ar-filled glovebox, yielding 5.0 M LiFSA/AN, 3.8 M LiFSA/PC, and 4.1 M LiFSA/DMF electrolytes. The water content of the prepared electrolytes was confirmed to be below 100 ppm by Karl Fischer titration. The experimentally determined densities of the electrolytes are summarized in Table S1.

*Measurements.* Electrochemical impedance spectroscopy (EIS) measurements were performed using a potentiostat/galvanostat (SP-150, BioLogic) over a frequency range of 20 mHz to 1.0 MHz. Impedance spectra were recorded in a three-electrode cell at 0.1 V vs. $Li/Li^+$ over a temperature range of 278–338 K. The working electrode was a commercially available graphite electrode (PIOTREK, Japan), while Li foil was used as both the counter and reference electrodes. The graphite electrode contained 95 wt% graphite, with an areal capacity of 1.6 mAh $cm^{-2}$ and an electrode area of 0.785 $cm^2$ (10 mm diameter). The obtained spectra were analyzed using an equivalent circuit model commonly employed for graphite electrodes undergoing $Li^+$ insertion reactions (shown in Figure S1a) to determine the charge-transfer resistance ($R_{ct}$). The $R_{ct}$ obtained from EIS analysis was converted to the rate constant ($k$) based on the Butler–Volmer equation. The activation thermodynamic parameters, $\Delta H^‡$, $\Delta S^‡$, and $\Delta G^‡$ ($= \Delta H^‡ - T\Delta S^‡$), were then determined using the Eyring equation based on transition state theory. Details of the analysis are provided in the following section. For comparison, the apparent activation energy ($E_a$) was evaluated from the temperature dependence of $R_{ct}$ using the Arrhenius relationship, i.e., $1/R_{ct} \propto A\exp(-E_a/RT)$, where $T$, $A$, and $R$ denote the

temperature, pre-exponential factor, and gas constant, respectively. $E_a$ was obtained from the slope of the $\log(1/R_{ct})$ vs. $1/T$ plot. Cyclic voltammetry (CV) was performed using a potentiostat (HZ-5000, Hokuto Denko) in a three-electrode beaker cell equipped with a Pt working electrode (2.25 $cm^2$) and lithium foil counter (1.2 $cm^2$) and reference (1.0 $cm^2$) electrodes. The electrolytes contained 1 mM ferrocene (Fc, Tokyo Chemical Industry Co., Ltd.) as a redox probe. CV measurements were conducted at a scan rate of 5.0 mV $s^{-1}$.

*MD simulations.* All-atom molecular dynamics (MD) simulations were performed using the GROMACS 2021.7 package under NPT ensemble conditions controlled by a Nosé–Hoover thermostat at 298 K and 1 atm.[1-2] The detailed simulation procedures were similar to those reported in our previous studies.[3-5] The system compositions (i.e., the numbers of $Li^+$, $FSA^-$, solvent molecules) and the equilibrium cell lengths are summarized in Table S1. The total simulation time was 15.0 ns. After equilibration for the first 10 ns, configurations collected at 0.1 ps intervals during the final 1.0 ns were used to calculate the X-ray-weighted structure factors and radial distribution functions, $S^{MD}(q)$ and $G^{MD}(r)$, respectively. The force fields used in the simulations were based on the CLaP model for $FSA^-$ and the OPLS-AA force field for AN, PC, and DMF, including intermolecular (Lennard–Jones and Coulombic) and intramolecular (bond stretching, angle bending, and torsional) interactions.[6-12] The Lennard–Jones parameters for $Li^+$ were adopted from a previous study on Li salts in carbonate-based solvents.[13] The partial atomic charges were assigned as follows. For AN, the charges were determined using the same procedure as in our previous study,[14] namely, from DFT calculations using the ChelpG method at the MP2/cc-pVTZ(-f)//HF/6-31G* level. For PC and DMF, literature charge parameters were adopted without modification.[7, 11] The partial charges of $Li^+$ and $FSA^-$ were scaled to account for the effect of strong ion–ion interactions in highly concentrated electrolytes. In our previous study, we demonstrated that a charge-scaling factor

of $f$ = 0.6 provides good agreement between simulated and experimental radial distribution functions.[12, 15] Accordingly, the same scaling factor was employed in the present study.

**Determination of activation thermodynamic parameters.**

The charge-transfer reaction at the electrode/electrolyte interface can be described by the Butler–Volmer equation, in which the current density ($i$) is expressed as a function of the overpotential ($\eta$):[16-17]

$$i = i_0\left[\exp\left(\frac{\alpha nF\eta}{RT}\right) - \exp\left(-\frac{(1-\alpha)nF\eta}{RT}\right)\right] \qquad (1),$$

where $i_0$ is the exchange current density, $\alpha$ is the charge-transfer coefficient, $n$ is the number of transferred electrons involved in the reaction, $F$ is the Faraday constant, $R$ is the gas constant, and $T$ is the absolute temperature. Electrochemical impedance spectroscopy measurements were performed at a fixed potential at 0.1 V vs. $Li/Li^+$, with a small AC perturbation of several mV superimposed on the applied potential. Under these conditions, the electrode reaction remains close to equilibrium, and the measured current response reflects the local differential response around the operating potential. Consequently, the Butler–Volmer equation can be linearized, allowing the charge-transfer resistance and related kinetic parameters to be quantitatively evaluated. Under the small-overpotential condition ($\eta \rightarrow 0$), the Butler–Volmer equation can be linearized using a first-order Taylor expansion. Because $\alpha + (1 - \alpha) = 1$, eq (1) reduces to

$$i \approx i_0 \frac{nF}{RT}\eta \qquad (2).$$

Thus, in this regime, the current response is proportional to the overpotential, and the proportionality constant is determined by the exchange current density $i_0$. The charge-transfer resistance ($R_{ct}$) is defined as

$$R_{ct} = \left(\frac{\partial \eta}{\partial i}\right)_{\eta \rightarrow 0} = \frac{RT}{nFi_0} \qquad (3).$$

Accordingly, the $i_0$ was calculated from the experimentally determined $R_{ct}$ values at each temperature. The $i_0$ is related to the rate constant ($k$) and the concentration of the reacting species ($C$) according to $i_0 = nFkC$ (eq. 4). Combining eqs (3) and (4) yields

$$k = \frac{RT}{n^2F^2CR_{\mathrm{ct}}} \qquad (5).$$

Thus, the rate constant $k$ was obtained from the experimentally determined charge-transfer resistance $R_{ct}$.

The temperature dependence of $k$ was analyzed using the Eyring equation based on transition-state theory:[18-19]

$$k = \frac{\kappa k_{\mathrm{B}}T}{h}\exp(-\frac{\Delta G^{\ddagger}}{RT}) = \frac{\kappa k_{\mathrm{B}}T}{h}\exp(-\frac{\Delta H^{\ddagger}}{RT})\exp(\frac{\Delta S^{\ddagger}}{R}) \qquad (6),$$

where $k_B$ is the Boltzmann constant, $h$ is the Planck constant, and $\kappa$ is the transmission coefficient. In the present study, $\kappa$ was assumed to be unity ($\kappa = 1$). Rearrangement of eq (6) gives

$$\ln(\frac{k}{T}) = -\frac{\Delta H^{\ddagger}}{R}\frac{1}{T} + \frac{\Delta S^{\ddagger}}{R} + \ln(\frac{k_B}{h}) \qquad (7).$$

Therefore, a plot of $\ln(k/T)$ versus $1/T$ (Eyring plot) yields a straight line, from which the activation enthalpy ($\Delta H^{\ddagger}$) and activation entropy ($\Delta S^{\ddagger}$) were determined from the slope and intercept, respectively. The activation Gibbs energy was subsequently calculated according to $\Delta G^{\ddagger} = \Delta H^{\ddagger} - T\Delta S^{\ddagger}$.

**Table S1.** Electrolyte compositions (Li salt concentration, $c_{\mathrm{Li}}$ and salt-to-solvent molar ratio, Li:X), number of LiFSA ion pairs and solvent molecules used in the MD simulation, simulation box length, and simulated and experimental densities (*d*) for 5.0 M LiFSA/AN, 3.8 M LiFSA/PC, and 4.1 M LiFSA/DMF electrolytes.

| Sample | $c_{\mathrm{Li}}$ / M | Li:X | Li–FSA | Solvent | Box length /Å | $d$ / g cm$^{-3}$ | |
|---|---|---|---|---|---|---|---|
| | | | | | | MD | Exp.[a] |
| LiFSA/AN | 5.0 | 1:2.0 | 1367 | 2733 | 75.0 | 1.444 | 1.342 |
| LiFSA/PC | 3.8 | 1:2.0 | 833 | 1667 | 70.5 | 1.547 | 1.500 |
| LiFSA/DMF | 4.1 | 1:2.0 | 880 | 1770 | 70.8 | 1.523 | 1.365 |

[a] Experimental values.

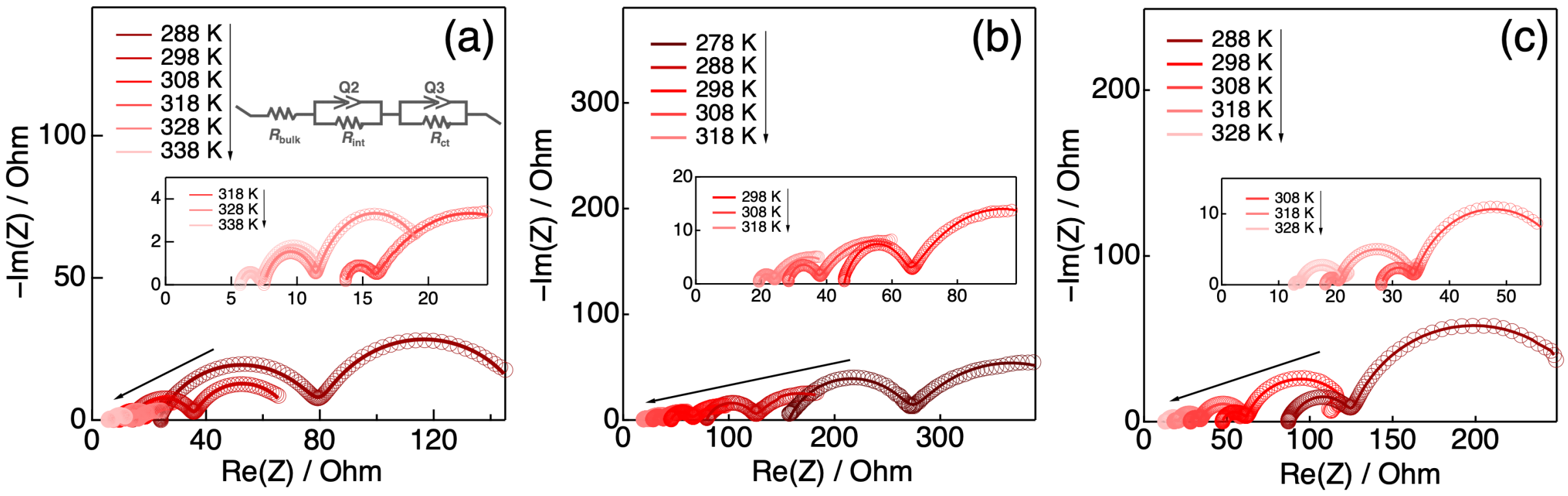


**Figure S1.** Temperature-dependent Nyquist plots of the graphite electrode in highly concentrated (a) LiFSA/AN, (b) LiFSA/PC, and (c) LiFSA/DMF electrolytes measured at 0.1 V vs. Li/Li$^+$. Open circles represent the experimental data, while solid lines represent the fitting results obtained using the equivalent circuit model shown in the inset. The inset in each panel shows an enlarged view of the high-temperature Nyquist plots for clarity. Measurements were performed in a three-electrode cell with graphite as the working electrode and Li foil as both the counter and reference electrodes.

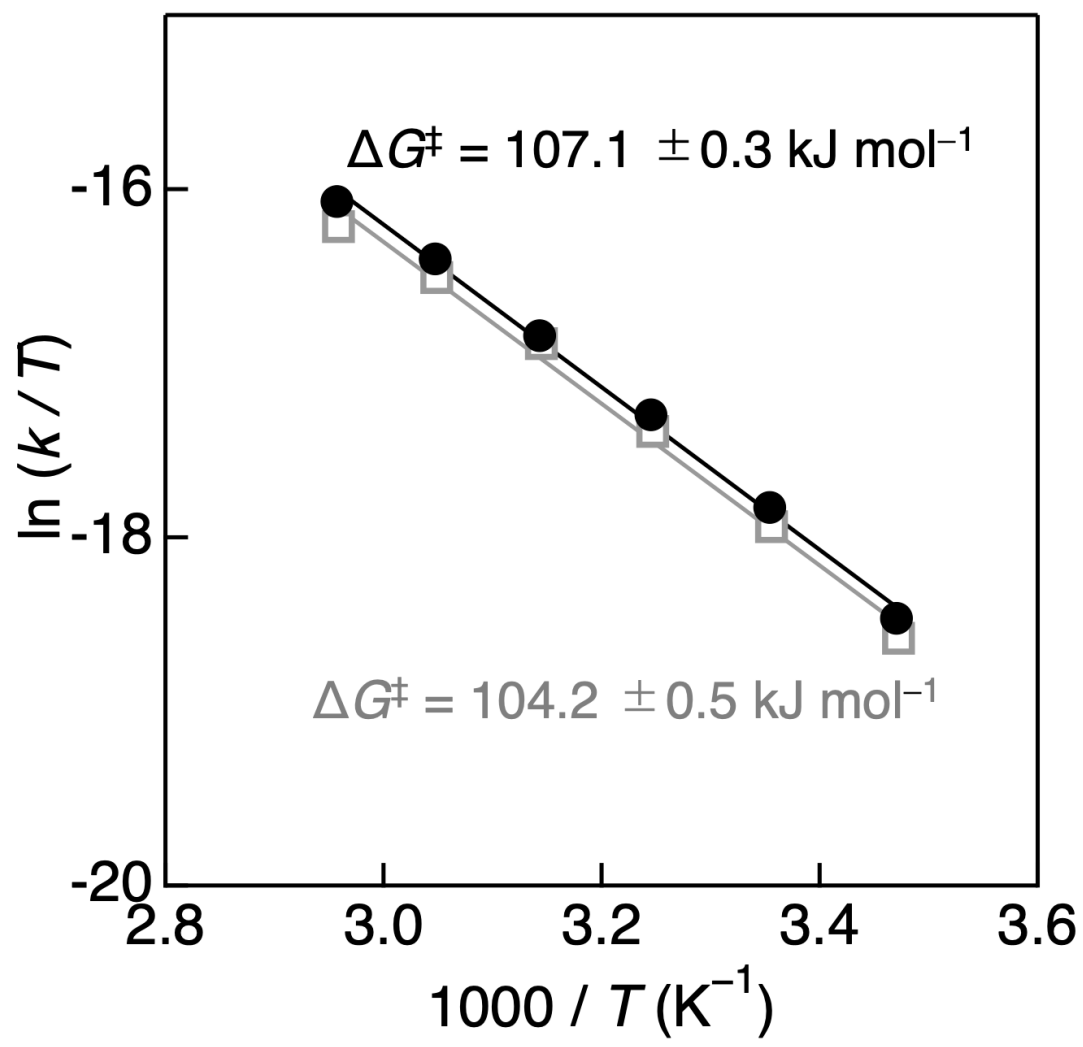


**Figure S2.** Eyring plots for $Li^+$ insertion into graphite in the 5.0 M LiFSA/AN electrolyte measured at 0.1 V vs. $Li/Li^+$. Black circles represent measurements using a pristine graphite electrode, whereas gray squares represent additional measurements using a graphite electrode pre-formed with an EC-derived solid electrolyte interphase (SEI).

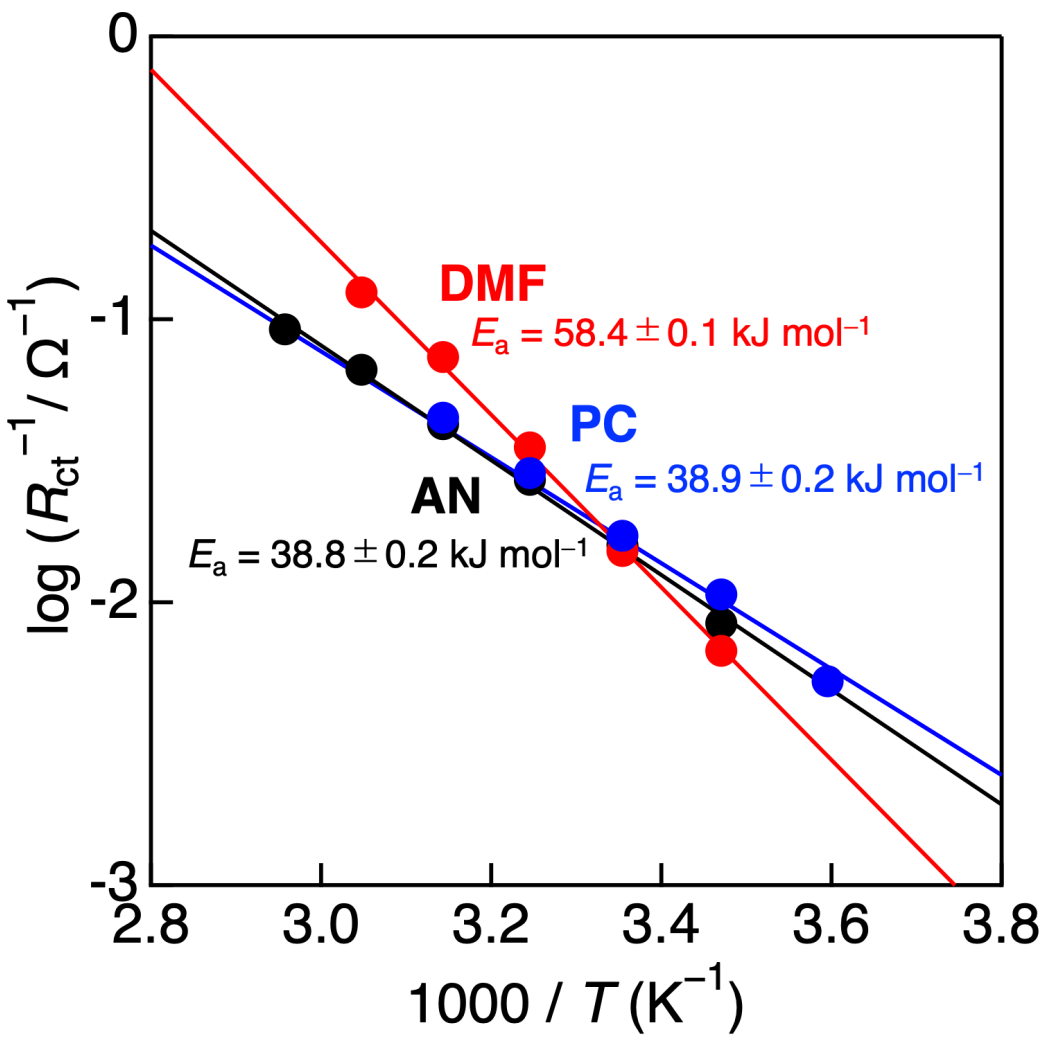


**Figure S3.** Arrhenius plots of $R_{ct}^{-1}$ for the graphite electrode in highly concentrated LiFSA/AN (black), LiFSA/PC (blue), and LiFSA/DMF (red) electrolytes measured at 0.1 V vs. $Li/Li^+$. Solid lines represent linear fits used to determine the apparent activation energy ($E_a$).

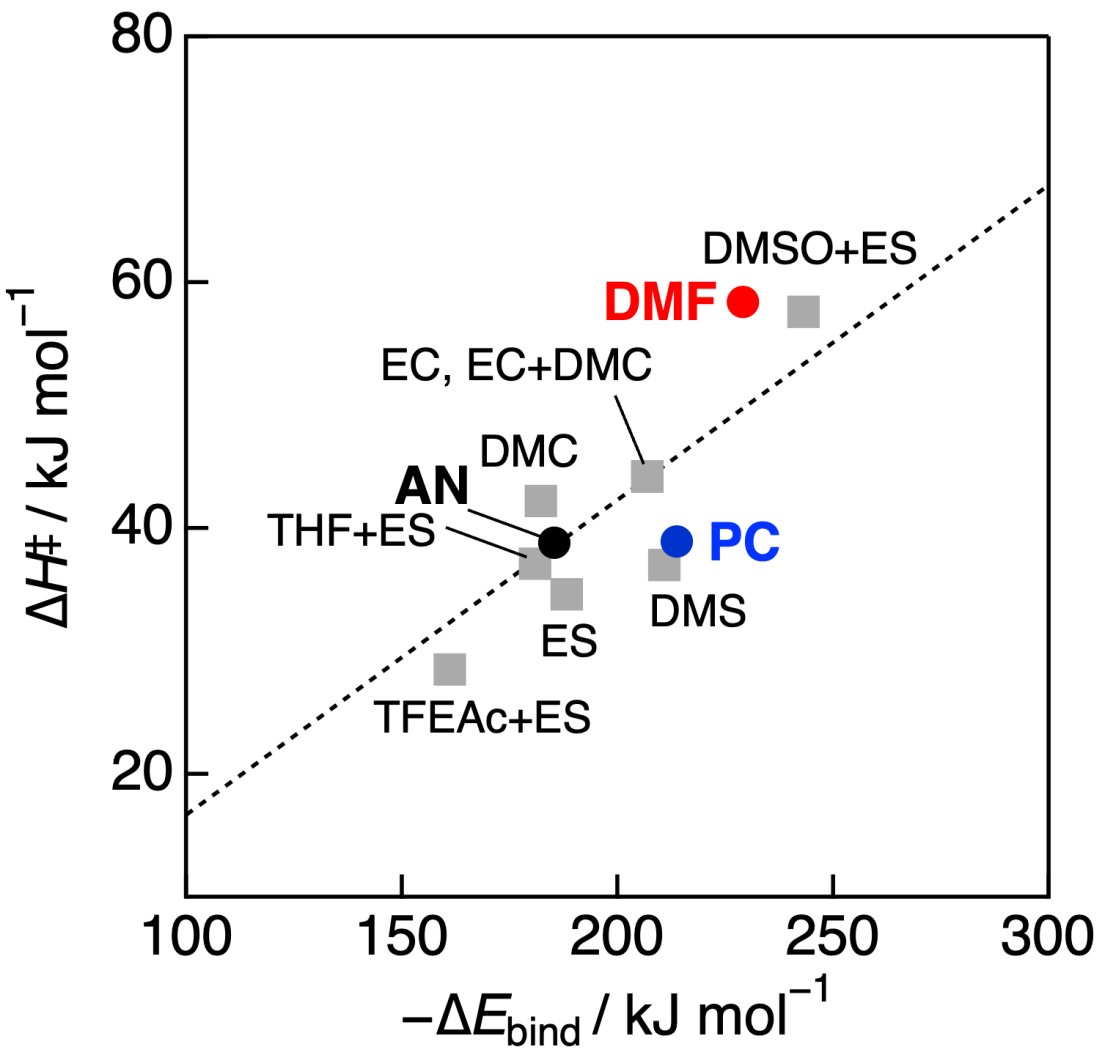


**Figure S4.** Correlation between the activation enthalpy ($\Delta H^{\ddagger}$) and the $Li^+$–solvent binding energy ($-\Delta E_{\mathrm{bind}}$) calculated by DFT for highly concentrated LiFSA/X electrolytes. Circles represent the present LiFSA/AN (black), LiFSA/PC (blue), and LiFSA/DMF (red) electrolytes, while gray squares represent literature data for TFEAc+ES, THF+ES, DMC, ES, EC, EC+DMC, DMS, and DMSO+ES electrolytes.[14]

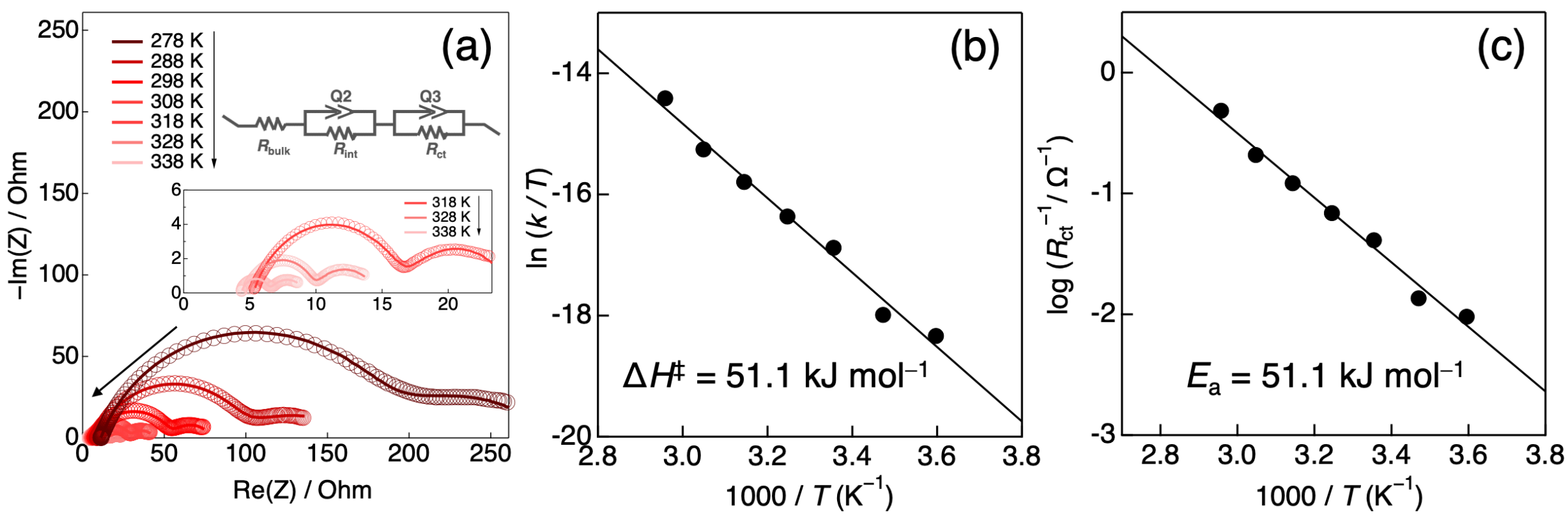


**Figure S5.** (a) Temperature-dependent Nyquist plots, (b) Eyring plot [$\ln(k/T)$ vs $1/T$], and (c) Arrhenius plot [$\log(1/R_{\mathrm{ct}})$ vs $1/T$] for the $Li^+$ insertion into graphite in 1.0 M LiFSA/EC+DMC electrolyte, measured at a fixed potential of 0.1 V vs $Li/Li^+$.

The impedance spectra exhibited two semicircles in the high- and low-frequency regions, and the diameter of the low-frequency semicircle, corresponding to the charge-transfer resistance ($R_{\mathrm{ct}}$) at approximately 4 Hz, decreased with increasing temperature (Figure S5a). The spectra were well reproduced using the equivalent-circuit model, allowing separation of the interfacial resistance ($R_{\mathrm{int}}$) and $R_{\mathrm{ct}}$ at each temperature. From the slope and intercept of the Eyring plot (Figure S5b), $\Delta H^{\ddagger}$ and $T\Delta S^{\ddagger}$ at 298 K were determined to be 51.1 and −49.9 kJ mol$^{-1}$,

respectively. Notably, the obtained $\Delta H^{\ddagger}$ value agrees well with the activation energy ($E_a$) determined from the Arrhenius plot (Figure S5c), yielding $E_a$ = 51.1 kJ mol$^{-1}$.

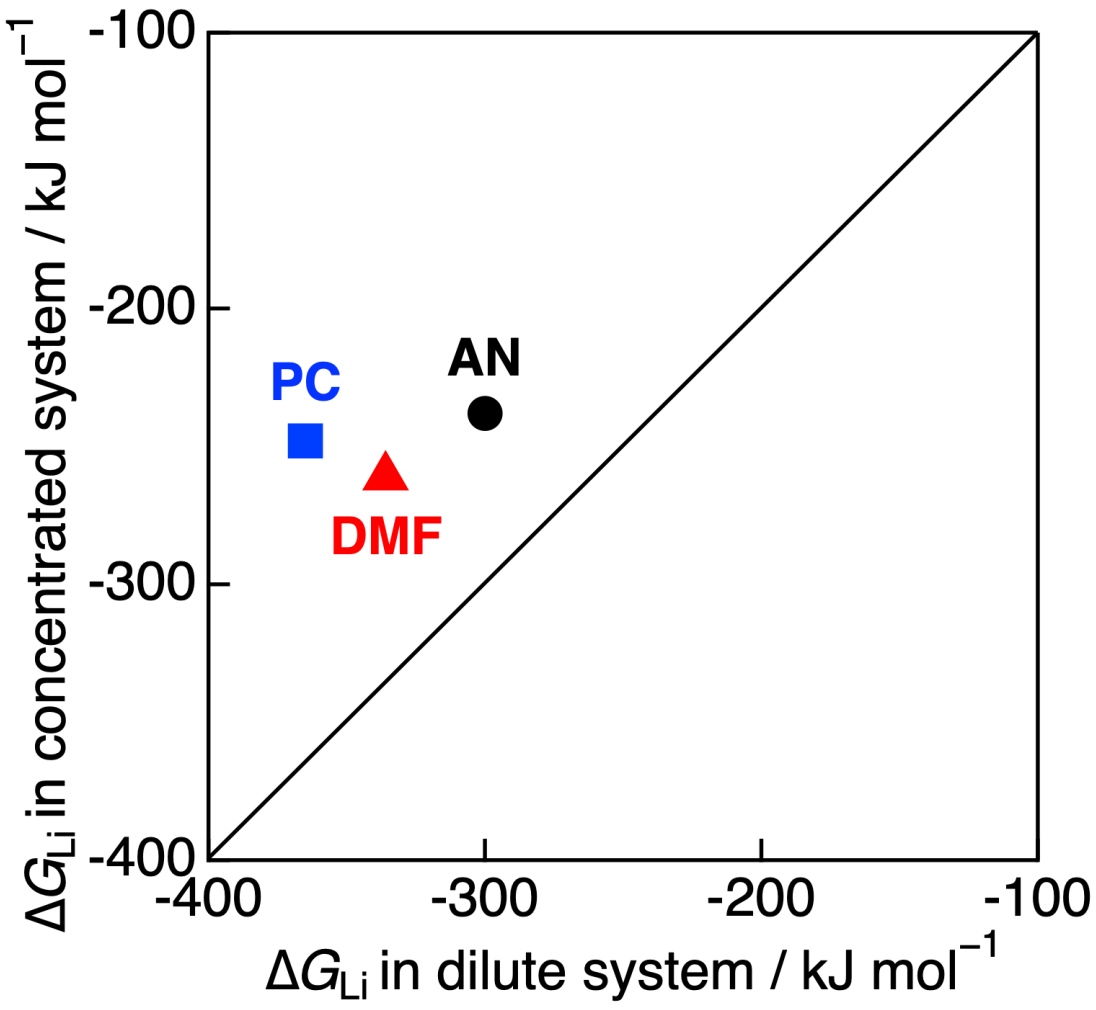


**Figure S6.** Comparison of $\Delta G_{Li}$ values in dilute[20] and highly concentrated electrolytes for LiFSA/AN (black circle), LiFSA/PC (blue square), and LiFSA/DMF (red triangle).

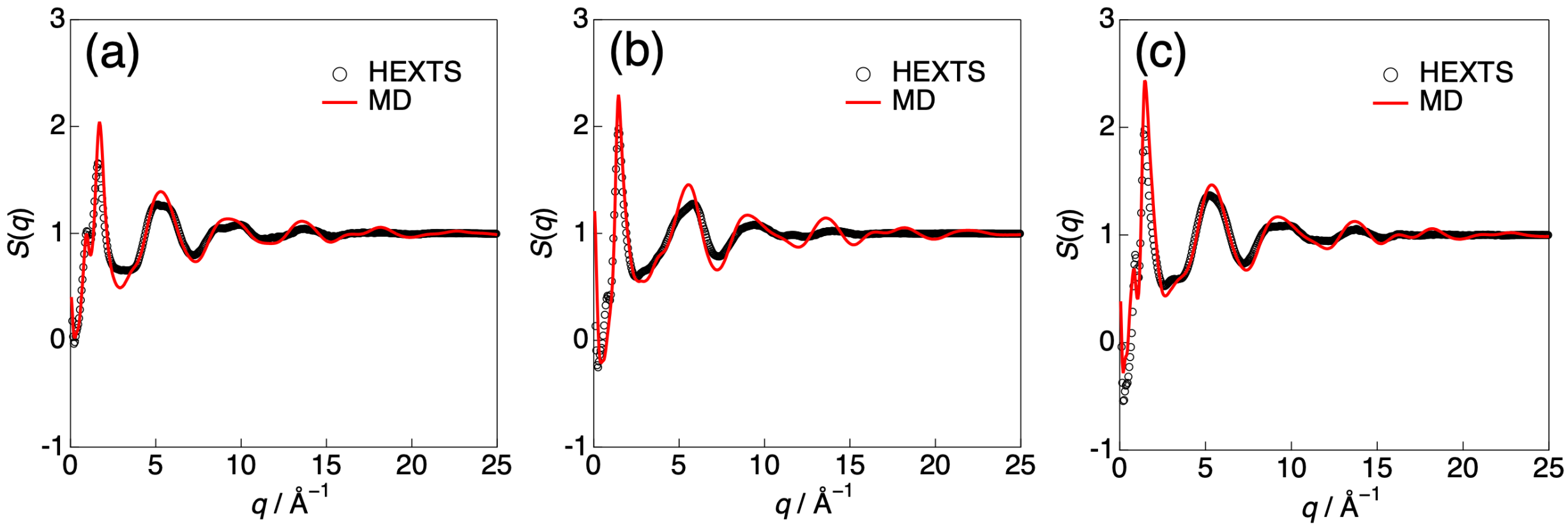


**Figure S7.** X-ray structure factors, $S(q)$ obtained from HEXTS experiments (open circles) and MD simulations (solid lines) for highly concentrated (a) LiFSA/AN, (b) LiFSA/PC, and (c) LiFSA/DMF electrolytes.

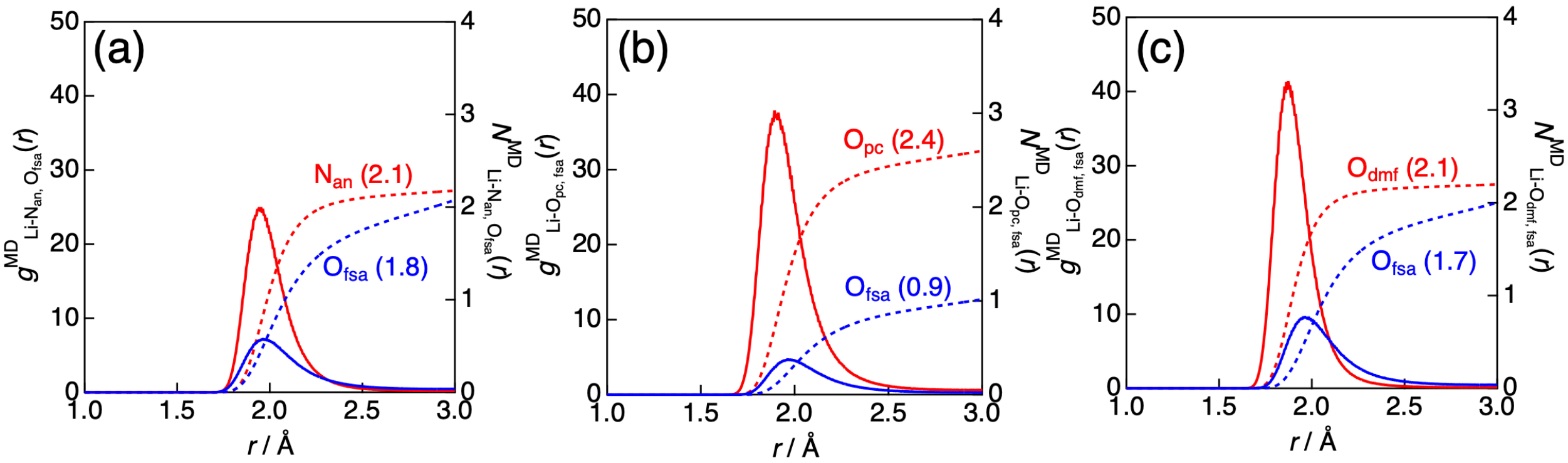


**Figure S8.** Pair distribution functions [$g^{MD}(r)$, solid lines; left axis] between $Li^+$ and the donor atoms of the solvent molecules (N in AN and O in PC and DMF, red) and between $Li^+$ and O atoms of $FSA^-$ (blue) for highly concentrated (a) LiFSA/AN, (b) LiFSA/PC, and (c) LiFSA/DMF electrolytes. The corresponding integrated coordination numbers [$N^{MD}(r)$, dashed lines; right axis] are also shown.

**References.**


(1) Nosé, S. A molecular dynamics method for simulations in the canonical ensemble. *Mol. Phys.* **1984,** *52*, 255-268.

(2) Hoover, W. G. Canonical dynamics: Equilibrium phase-space distributions. *Phys. Rev. A* **1985,** *31*, 1695-1697.

(3) Fujii, K.; Ueki, T.; Hashimoto, K.; Kobayashi, Y.; Kitazawa, Y.; Hirosawa, K.; Matsugami, M.; Ohara, K.; Watanabe, M.; Shibayama, M. Microscopic Structure of Solvated Poly(benzyl methacrylate) in an Imidazolium-Based Ionic Liquid: High-Energy X-ray Total Scattering and All-Atom MD Simulation Study. *Macromolecules* **2017,** *50*, 4780-4786.

(4) Sawayama, S.; Kawaguchi, T.; Fujii, K. Role of Anion Flexibility on Graphite Electrode Reactions in Bis(fluorosulfonyl)amide-Based Ionic Liquid Electrolytes for Lithium-Ion Batteries. *J. Phys. Chem. C* **2024,** *128*, 19134-19141.

(5) Sawayama, S.; Kondou, S.; Ueno, K.; Fujii, K. Conformational Flexibility of Solvent Molecules Enables Li-Ion Hopping in Highly Concentrated Electrolytes. *J. Phys. Chem. B* **2025,** *129*, 7995-8001.

(6) Cornell, W. D.; Cieplak, P.; Bayly, C. I.; Gould, I. R.; Kenneth M. Merz, J.; Ferguson, D. M.; Spellmeyer, D. C.; Fox, T.; Caldwell, J. W.; Kollman, P. A. A Second Generation Force Field for the Simulation of Proteins, Nucleic Acids, and Organic Molecules. *J. Am. Chem. Soc.* **1995,** *117*, 5179-5197.

(7) Jorgensen, W. L.; Maxwell, D. S.; Tirado-Rives, J. Development and Testing of the OPLS All-Atom Force Field on Conformational Energetics and Properties of Organic Liquids. *J. Am. Chem. Soc.* **1996,** *118*, 11225-11236.

(8) Price, M. L. P.; Ostrovsky, D.; Jorgensen, W. L. Gas-Phase and Liquid-State Properties of Esters, Nitriles, and Nitro Compounds with the OPLS-AA Force Field. *J. Comput. Chem.* **2001,** *22*, 1340-1352.

(9) Shimizu, K.; Almantariotis, D.; Gomes, M. F. C.; Pádua, A. A. H.; Lopes, J. N. C. Molecular Force Field for Ionic Liquids V: Hydroxyethylimidazolium, Dimethoxy-2-Methylimidazolium, and Fluoroalkylimidazolium Cations and Bis(Fluorosulfonyl)Amide, Perfluoroalkanesulfonylamide, and Fluoroalkylfluorophosphate Anions. *J. Phys. Chem. B* **2010,** *114*, 3592-3600.

(10) Cui, S.; de Almeida, V. F.; Khomami, B. Molecular dynamics simulations of tri-*n*-butyl-phosphate/*n*-dodecane mixture: thermophysical properties and molecular structure. *J. Phys. Chem. B* **2014,** *118*, 10750-10760.

(11) Vasudevan, V.; Mushrif, S. H. Force field parameters for *N*,*N*-Dimethylformamide (DMF) revisited: Improved prediction of bulk properties and complete miscibility in water. *J. Mol. Liquids* **2015,** *206*, 338-342.

(12) Sawayama, S.; Morinaga, A.; Mimura, H.; Morita, M.; Katayama, Y.; Fujii, K. Fluorophosphate-Based Nonflammable Concentrated Electrolytes with a Designed Lithium-Ion-Ordered Structure: Relationship between the Bulk Electrolyte and Electrode Interface Structures. *ACS Appl. Mater. Interfaces* **2021,** *13*, 6201-6207.

(13) Soetens, J.-C.; Millot, C.; Maigret, B. Molecular Dynamics Simulation of $Li^+BF_4^-$ in Ethylene Carbonate, Propylene Carbonate, and Dimethyl Carbonate Solvents. *J. Phys. Chem. A* **1998,** *102*, 1055-1061.

(14) Sawayama, S.; Matsugami, M.; Fujii, K. Direct Molecular Evidence for Desolvation-Controlled Lithium-Ion Insertion at Graphite Electrodes in Highly Concentrated Electrolytes. *J. Phys. Chem. Lett.* **2025,** *16*, 9334-9338.

(15) Sogawa, M.; Sawayama, S.; Han, J.; Satou, C.; Ohara, K.; Matsugami, M.; Mimura, H.; Morita, M.; Fujii, K. Role of Solvent Size in Ordered Ionic Structure Formation in Concentrated Electrolytes for Lithium-Ion Batteries. *J. Phys. Chem. C* **2019,** *123*, 8699-8708.

(16) Butler, J. A. V.; Sc., M. Studies in heterogeneous equilibria. Part II.—The kinetic interpretation of the nernst theory of electromotive force. *Trans. Faraday Soc.* **1924,** *19*, 729-733.

(17) Bard, A. J.; Faulkner, L. R. *Electrochemical methods: fundamentals and applications (2nd ed.)*, John Wiley & Sons: New York, 2011.

(18) Eyring, H. The Activated Complex and the Absolute Rate of Chemical Reactions. *Chem. Rev.* **1935,** *17*, 65-77.

(19) Swaddle, T. W. Activation parameters and reaction mechanism in octahedral substitution. *Coord. Chem. Rev.* **1974,** *14*, 217-268.

(20) Marcus, Y. *Ion Properties*, Marcel Dekker: New York, 1997.
